# Pemphigoid Gestationis after Spontaneous Expulsion of a Massive Complete Hydatidiform Mole

**DOI:** 10.1155/2013/267268

**Published:** 2013-09-08

**Authors:** Naoki Matsumoto, Marie Osada, Kou Kaneko, Ken Ohara, Daito Noguchi, Haruhiko Udagawa, Nagazumi Suzuki, Chieko Matsumoto, Sachio Takahashi

**Affiliations:** ^1^Department of Obstetrics and Gynecology, Fukaya Red Cross Hospital, 5-8-1 Kamishibachonishi, Fukaya City, Saitama 366-0052, Japan; ^2^Department of Obstetrics and Gynecology, Tatedebari Sato Hospital, 96 Wakamatsucho, Takasaki City, Gunma 370-0836, Japan; ^3^Department of Pathology, Fukaya Red Cross Hospital, 5-8-1 Kamishibachonishi, Fukaya City, Saitama 366-0052, Japan; ^4^Department of Obstetrics and Gynecology, Saitama Medical Center, Saitama Medical University, 1981 Kamoda, Kawagoe City, Saitama 350-8550, Japan

## Abstract

Pemphigoid gestationis (PG) is a rare, perinatal, autoimmune, and blistering dermatosis. Only few cases of PG involving hydatidiform moles have been reported. Complete hydatidiform moles are usually evacuated by dilatation and curettage. We report a patient with a massive complete hydatidiform mole that underwent spontaneous expulsion; she subsequently developed PG. A 19-year-old unmarried nulligravid woman was referred to our hospital following excessive vaginal bleeding after an uncertain amenorrheal period. The patient presented with preshock vital signs, severe anemia, and a positive urine pregnancy test. Imaging examinations revealed a massive intrauterine mass (19 × 15 × 10 cm), suggesting a complete hydatidiform mole. She was hospitalized and treated with blood transfusion. Sixteen hours after hospitalization, the massive molar mass underwent spontaneous expulsion and bleeding ceased. Three days after the expulsion, she developed pruritic skin lesions including papules, erythemas, and bullae, which spread over her entire body. Skin biopsy revealed PG and subepidermal blister formation and linear complement C3 deposition along the basement membrane zone, and the serum anti-BP180 antibody level was found to be high on measurement. She was effectively treated with 50 mg/day of oral prednisolone. Her skin lesions disappeared, leaving pigmentation.

## 1. Introduction

Recently, in Japan, almost all pregnancies, irrespective of normal or abnormal, are examined during the early gestational weeks. When a hydatidiform mole in the uterus is suspected following an imaging examination, such as ultrasonography, it is usually evacuated by dilatation and curettage (D&C) for histopathological diagnosis and treatment. However, massive moles are occasionally difficult to remove by D&C. 

 Pemphigoid gestationis (PG), which has previously been called “herpes gestationis,” is a rare, autoimmune, self-limiting, and blistering dermatosis associated with pregnancy [1]. Its incidence is considered to be 1 in 50,000 to 60,000 pregnancies [2]. Hydatidiform moles occur in about 1 in 1,200 to 1,500 pregnancies [3, 4]. Therefore, PG in hydatidiform mole patients is considered extremely rare.

 We report a patient who presented with a massive complete hydatidiform mole that underwent spontaneous expulsion; she subsequently developed PG. We report a summary of our patient from obstetrical and gynecological aspects.

## 2. Case Presentation

**Figure 1:**
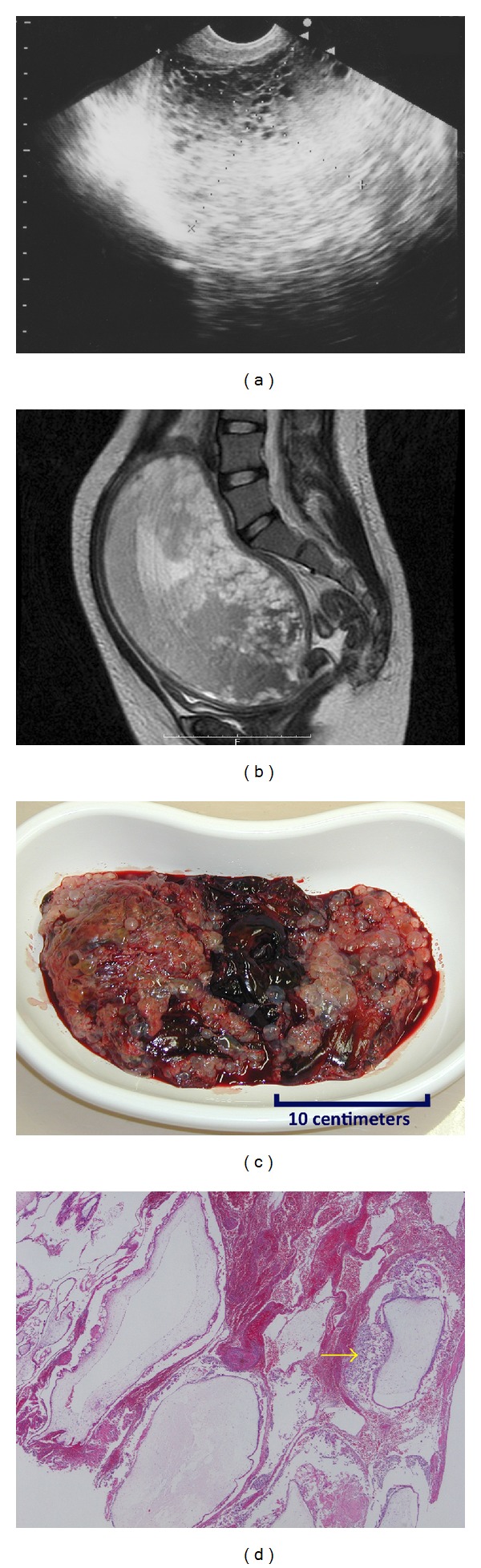
Images of the complete hydatidiform mole. (a) Transvaginal ultrasonography (sagittal). (b) The magnetic resonance image (T2 weighted, sagittal) shows a massive intrauterine mass (19 × 15 × 10 cm) with many small vesicles, no normal gestational sac, and no fetus. (c) The macroscopic image of the expelled mole that has a classical bunch of grapes appearance. (d) The microscopic image (hematoxylin and eosin staining) shows the edematous and swollen villi with circumferential trophoblastic proliferation (as the arrow indicates).

The patient was a 19-year-old unmarried nulligravid Japanese woman with no relevant past history. Her sexual partner was a 40-year-old Japanese man. On September 23, 2007, she was referred to the emergency care unit of Fukaya Red Cross Hospital, Saitama, Japan. Her chief complaint was excessive vaginal bleeding after an uncertain period of amenorrhea. She presented with preshock vital signs: a clear conscious state, no fever, a pulse rate of 124 bpm, and a blood pressure of 112/62 mmHg. Her hemoglobin concentration was 3.7 g/dL and a qualitative urine human chorionic gonadotropin (hCG) test was positive. Both ultrasonography (Figure 1(a)) and magnetic resonance imaging (Figure 1(b)) showed a massive intrauterine mass (19 × 15 × 10 cm) with many small vesicles, no normal gestational sac, and no fetus; these features strongly suggested the diagnosis of a complete hydatidiform mole. Other abnormal values of clinical laboratory tests are shown in Table 1. 

She was immediately hospitalized and treated with fluid infusion and blood transfusion. Furthermore, it was considered that D&C is necessary. However, the D&C posed a risk of more bleeding and perforation because the uterus was extremely enlarged and its muscular wall was very thin. Hence, we were reluctant to immediately perform a D&C. Her vital signs were stable, but vaginal bleeding continued intermittently. Therefore, we considered that we should carefully attempt a suction D&C using drugs that would prompt uterine contractions. Sixteen hours after hospitalization, the bleeding suddenly increased, followed by spontaneous expulsion of a large mass. The uterus immediately contracted to the size of an orange, reducing further bleeding. Macroscopic (Figure 1(c)) and microscopic (Figure 1(d)) examinations of the mass confirmed the diagnosis of a complete hydatidiform mole. She underwent suction D&C after the spontaneous expulsion to ensure complete evacuation of the mole. Her mild renal dysfunction had been gradually improving without proteinuria and oliguria.

**Figure 2:**
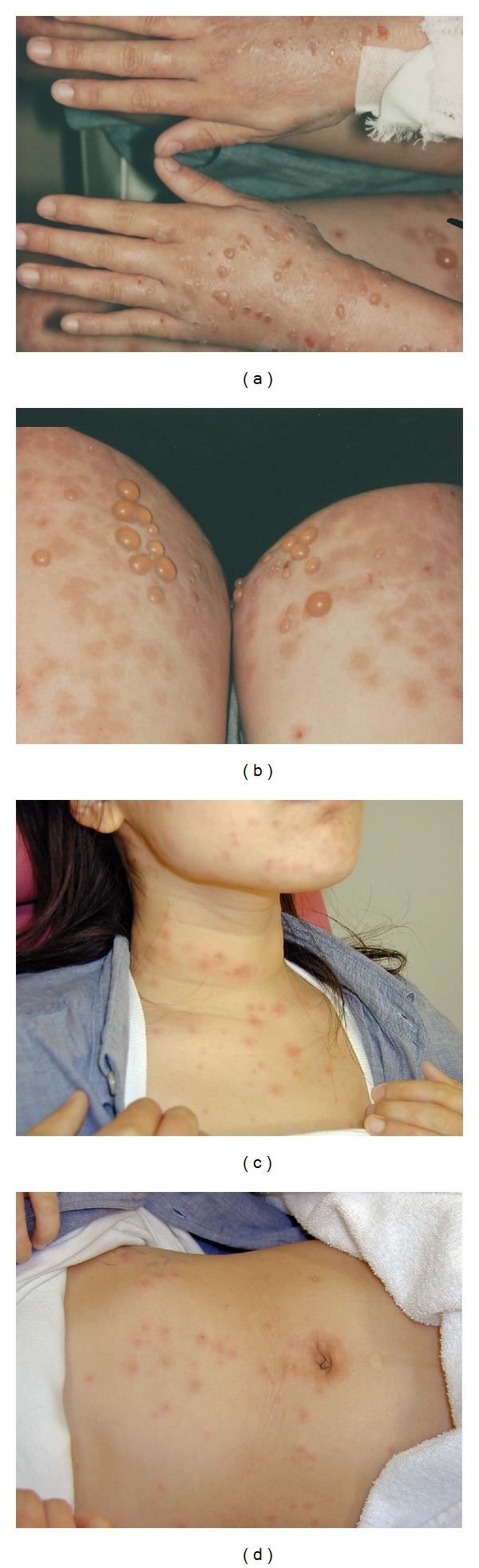
Macroscopic skin lesions including many tense bullae and edematous erythemas. (a) On the hands. (b) On the femurs. (c) On the neck. (d) On the abdomen.

**Figure 3:**
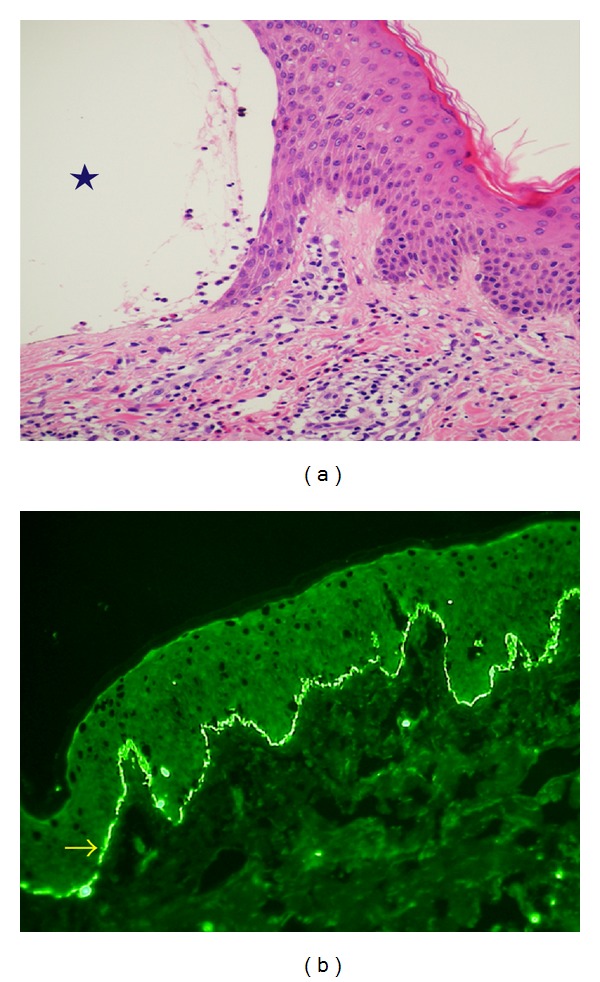
Biopsy specimens of the skin lesions. (a) The hematoxylin and eosin staining shows subepidermal blister formation (as the star indicates) with cellular infiltration composed mainly of lymphocytes with numerous eosinophils. (b) Direct immunofluorescence shows linear complement C3 deposition (as the arrow indicates) along the basement membrane zone.

 Three days after expulsion of the mole, she developed pruritic skin lesions. The lesions included papules and erythemas, which appeared on the dorsum of her hands and the anterior part of the chest at first. She was initially treated with an oral antihistamine and a local corticosteroid, but the treatment was not effective. Some of the lesions soon transformed into vesicles and bullae, which subsequently spread over her entire body (Figures 2(a)–2(d)). We further decided to consult dermatologists. A skin biopsy revealed subepidermal blister formation (Figure 3(a)). Linear complement C3 deposition was observed along the basement membrane zone on direct immunofluorescence analysis (Figure 3(b)). Additional laboratory tests were performed to assess the dermatosis (Table 1). Eosinophilia appeared after the eruption. Her serum immunoglobulin E level and anti-BP180-NC16a antibody index (Mesacup BP180 Test, Medical & Biological Laboratories Co., LTD) [5, 6] were very high (Table 1). Therefore, the skin lesions were diagnosed as PG. She was transferred to the Jichi Medical University Hospital, Tochigi, Japan, because of geographical reasons. She further received 30 mg/day (0.6 mg/kg/day) oral prednisolone; however, new eruptions occurred after a few days. Therefore, the dose was increased to 50 mg/day (1 mg/kg/day), which proved to be very effective. Prednisolone treatment was gradually reduced and stopped after 10 weeks. 

 The patient's serum hCG level propitiously decreased and became undetectable 12 weeks after expulsion of the mole. Sixteen weeks after expulsion, the hCG level remained undetectable and the skin lesions had disappeared without recurrence but leaving pigmentation. However, she did not visit the hospital after the above-mentioned consecutive treatments, so no information regarding her further course was available. 

## 3. Discussion

We have shown that spontaneous expulsion of a large molar mass does occur. In Japan, recently, almost all pregnancies are examined in the early gestational weeks. A suspected hydatidiform mole will usually be evacuated by D&C as early as possible [3]. No randomized controlled studies exist on the method of evacuation of molar diseases. However, D&C is considered to be the preferred treatment for the evacuation of moles; moreover, suction D&C is preferred over sharp D&C [7, 8]. Medical induction of labor with oxytocin or prostaglandins is not recommended [8]. In our patient, we were apprehensive about performing a D&C because the risk of bleeding and perforation was considered very high. Fortunately, the massive mole underwent spontaneous expulsion, and further bleeding was prevented. Because of this unanticipated spontaneous event, we could avoid the high risk D&C. 

 We have discussed here the practical, diagnostic, and therapeutic aspects of PG. PG is a perinatal, autoimmune, pruritic, and vesiculobullous skin disorder, pathophysiologically resembling bullous pemphigoid. PG also occurs in moles [9–12] and choriocarcinomas [13, 14]. PG should be differentiated from several perinatal pruritic dermatoses such as pruritic urticarial papules and plaques of pregnancy. The three chief symptoms required for diagnosis of PG are as follows: tense subepidermal blisters, complement C3 and/or immunoglobulin G deposition along the basement membrane zone on direct immunofluorescence, and serum anti-BP180-NC16 antigen [2]. In the treatment of PG, oral corticosteroids are standard [2]. Prednisolone is usually started at a dose of 20–40 mg/day, and higher doses may be necessary in severe cases [1].

 We report an extremely rare case, in which the patient presented with both complete hydatidiform mole and PG. Four previous cases in which PG coincided with a hydatidiform mole have been reported [9–12]. A summary of these cases and our patient is presented in Table 2. The incidence of molar diseases is rare and that of PG is even rarer; therefore, patients with both of these conditions are extremely rare. Prompt and precise diagnosis of PG remains challenging for obstetricians and gynecologists; however, we hope that this report will help them to diagnose and treat similar cases. 

## Figures and Tables

**Table 1:** Abnormal values of clinical laboratory tests.

Hospital day	Events	Laboratory tests and their values
1st	On admission	Hb 3.7 g/dL
		WBC 22,100/mL (Neu 62.6%, Eo 4.3%, Ly 26.8%)
		Serum creatinine 1.7 mg/dL, blood urea nitrogen 31 mg/dL

3rd	After blood transfusion and expulsion of the mole	Hb 6.9 g/dL
		Serum hCG 80,700 mIU/mL

13th	After diagnosis of PG and before oral prednisolone administration	WBC 20,600/mL (Neu 68.4%, Eo 14.4%, Ly 12.5%)
		Serum immunoglobulin E 3970 U/mL
		Anti-BP180-NC16a antibody index 360^†^

Eo: eosinophils; Hb: hemoglobin; hCG: human chorionic gonadotropin; Ly: lymphocytes; Neu: neutrophils; PG: pemphigoid gestationis; WBC: white blood cell count.

^†^The normal limit of this index is <9.

**Table 2:** Review of the literatures. Our patients and 4 previously reported molar cases which developed pemphigoid gestationis.

Year	Authors	Patient's age (y)	Previous gestations	DIF used for diagnosis	The first skin lesions were seen
1950	Tillman [9]	42	2	No	7 days after abortion
1974	Dupont [10]^†^	—	—	—	—
1975	Yasue [11]	53	4	Yes	Before D&C
1981	Tindall et al. [12]	28	5	Yes	3 days after D&C
2013	This report	19	0	Yes	3 days after spontaneous expulsion and D&C

D&C: dilatation and curettage; DIF: direct immunofluorescence.

All cases were complete hydatidiform moles.

^†^We could not obtain details of the literature.
